# Attitudes of oncology patients’ towards biospecimen donation for biobank research

**DOI:** 10.1186/s12885-024-12145-5

**Published:** 2024-03-27

**Authors:** Jan Domaradzki, Justyna Czekajewska, Dariusz Walkowiak

**Affiliations:** 1 Department of Social Sciences and Humanities, Poznan University of Medical Sciences, Rokietnicka 7, 60-806 Poznań, Poznań, Poland; 2 Department of Organization and Management in Health Care, Poznan University of Medical Sciences, Przybyszewskiego 39, 60-356, Poznań, Poland

**Keywords:** Biobanking, Cancer-related research, Cancer patients, Donation, Human biological material, Research Biobanks, Tissue donation

## Abstract

**Background:**

Since the biological material that remains after diagnostic and therapeutic procedures plays crucial role in biobank research, this study aims to explore cancer patients’ views on the donation of biospecimens for research purposes.

**Methods:**

548 oncology patients from two hospitals with oncology treatment units in Poznan, Poland, completed an anonymous, self-administered pen-and-paper questionnaire.

**Results:**

Although only 43.4% of patients had heard of biobanks, 93.1% declared themselves willing to donate. 71.1% of patients believed that doctors should ask patients to donate, and 60.9% that this should be done before the medical procedure. While 65% of patients were willing to donate any type of tissue that remained after a medical procedure, blood, saliva and hair were indicated most frequently. 40.5% of patients would donate their entire body after death and 21% would refuse. Patients’ support for biobanks was mainly driven by the desire to support science, help advance cancer research and altruism. Some respondents expected health information or medical treatment. The most common barriers for donation were physical distance, repeated examinations, concerns over the privacy and confidentiality of data and the commercial or unethical use of samples. Patients’ attitudes toward biobank donation seemed to be associated with age, education level, declared religiousness, a family history of genetically determined diseases and whether they were a blood donor.

**Conclusions:**

Although cancer patients’ lack of biobank awareness had no effect on their affirmative attitudes towards biobank research, there is a need to further increase patients’ support and overcome possible barriers that might hinder their willingness to donate.

## Background

Cancer-related genetic research has become an important area that can help to develop new diagnostic techniques and treatment approaches. While laboratory-based research on cancer may lead to the development of precision or personalised medicine, it takes a long time and requires vast numbers of biosamples from different patients. The role of biobanks in cancer research is therefore critical [1, 2]. Since biobanks collect biospecimens (e.g. blood, DNA, salvia, urine, cells, tissues, etc.) and annotated data (e.g. clinical, pathological, demographic, socio-economic, genealogical, lifestyle and environmental) from a large cohort of individuals [3–6], they allow the assessment of the risk these factors pose in the development of numerous chronic diseases, including cancer. As the main objective of biobanks is to collect, store, preserve and supply biological samples and relevant data for future use in research [4, 7, 8], they are also crucial for translational and clinical cancer-related research in oncology on the molecular mechanisms of cancer and cancer drug resistance, and also have the potential to lead to breakthroughs in precision oncology. Finally, since biobanks allow the sharing of biospecimens among researchers, biobanks and research institutions from other countries, they create an opportunity to further advance modern large-scale research in the field of cancer [1, 2, 9]. Many countries worldwide have made huge investments in the creation of local, state and national biobank infrastructures.

This is particularly important for countries with high incidences of cancer morbidity and mortality, such as Poland, where cancer is the second leading cause of death after cardiovascular diseases [10]. Apart from launching the National Cancer Strategy 2020–2030, i.e. the first national cancer plan, which seeks to improve prevention, early detection and the treatment of several types of cancer, and an increase in the five-year survival rates after cancer treatment in both women and men [11], in 2016 the Polish Biobanking Network (PBN) was created and operates within the European Biobanking and BioMolecular resources Research Infrastructure-European Research Infrastructure Consortium (BBMRI-ERIC) consortium [12]. Its main objective is to connect all Polish biobanks and facilitate their co-operation in the field of sharing of information about their biological material collections and facilitating communication between scientists and individual units in the country [13]. It is therefore expected that PBN will lead to an increase in the number of experiments and projects conducted and to an increase in the credibility of research carried out in Poland.

However, because biobanking in Poland is still in the early development, to date there are no specific legal regulations regarding biobanking. Moreover, there are no rules for using human biological material and clinical data for scientific purposes; nor there is any control over genetic testing in the country [14]. This is important, since biobank research raises important ethical, legal and social (ELSI) challenges related to, *inter alia* patients’ autonomy, data protection and confidentiality, control of information and sharing of biosamples, commercialization and profit sharing, which may affect people’s trust towards biomedical research and their willingness to donate^1^ to biobanks [15–20]. For that reason, the PBN plays a crucial role in the development and implementation of standards for procedures and management formulated according to the guidelines created by the International Society for Biological and Environmental Repositories (ISBER) which is a key organization in the activity and innovation for biobanking and the harmonization of scientific, technical, ethical and legal issues [21–23]. Consequently, although still Poland still has no legal regulations on biobanks or specific biobank act, in 2021 following international and European regulations and recommendations created by BBMRI-ERIC and ISBER quality standards for Polish biobanks have been created. These new guidelines for good practices encompass 15 distinct areas, covering aspects such as: organization and institution management, quality management, documentation and records, human resource management, ethical and legal considerations, supplies and materials management, devices, traceability, environmental and staff hygiene, technological processes and quality control, handling deviations and incompatible product/data or service, conducting audits, implementing improvements, fostering scientific cooperation, and ensuring safety [22, 24, 25].

The biological material remaining after diagnostic and therapeutic procedures constitutes an invaluable source of research information and may be used to conduct many research projects and further develop science and personalised medicine [4, 7, 26, 27]. However, even though from the biobank’s perspective broad consent for biobanking and future research is preferable, some research show that it is not optimal for the biobank participants, as they often opt for other types of consent, including one-time or study-specific consent [28–30]. Moreover, the consent to biobanking alone does not authorize in a broad sense to carry out any kind of research activities, but it is necessary to seek ethics committee approval. Thus, it is therefore important to ensure highest standards of operation and adequate funding, training, certification and the Quality Management System (QMS) [22, 24–27, 31]. As well as implementing and constantly improving QMS, cancer biobanks also require large numbers of different patients to submit biospecimens and personal health information for research purposes. To achieve this there is a need for better understanding the views of different stakeholders, including patients, who may share their human biological material (HBM) and associated health information for research purposes. Since many people, including patients, express many ethical, legal, and social concerns related to biobanking [28, 32–39], knowing their perspective may help recruit new donors to contribute to cancer research.

However, although the research on the people’s views on biobank research is growing rapidly, over the last decade only 25 research on cancer patients’ perspective have been conducted, according to the PubMed database [28, 32–48]. Similarly, while there are only a few studies assessing the views of the Poles on the attitudes towards biobanking and donation of HBM they focus on the general population [49–54], but there remains a shortage of research on the perception of biobank research among Polish cancer patients. This study therefore seeks to explore oncology patients’ views on the donation of biospecimens for research purposes, including: (1) awareness regarding biobank research, (2) willingness to donate (cancer) tissue remaining after medical procedure for research purposes, (3) motivation for (non)participation, (4) type of tissues they would be willing to donate for research purposes, and (5) factors associated with patients decisions to donate tissue for biobank research.

## Methods

### Study design

This study was designed to explore oncology patients’ views on tissue donation for research purposes. It presents data from a self-administered, anonymous, pen-and-paper survey on biobank awareness and the perception of biobank research among Polish cancer patients, their motivations for donation and reasons for non-participation.

### Participants and setting

A sample of 548 oncology patients was recruited from two hospitals in Poznan with oncology treatment units. The survey was collected between 1st February 2023 and 30th June 2023.

The following inclusion criteria were used: participants had to be at least 18 years of age, to have been diagnosed with cancer, to be willing to participate in the survey and to provide written informed consent before completing the survey.

### Research tool

Once a literature review on cancer patients’ perceptions on biospecimen donation for cancer research had been conducted the questionnaire used in this study was designed with the help of several experts from the fields of public health, medical sociology and bioethics in accordance with the guidelines of the European Statistical System [55]. The preliminary questionnaire was pilot tested using 20 cancer patients, resulting in the re-formulation of three questions. The final version of the questionnaire was approved by the Poznan University of Medical Sciences Bioethics Committee.

The questionnaire itself consisted of four sections, each corresponding to a specific aspect of tissue donation. The first asked questions concerning cancer patients’ biobank awareness (whether they had heard of biobanks, their willingness to donate cancer tissue remaining after a medical procedure to a biobank, patients’ preferences on who should ask patients to donate and when). The second section included questions regarding patients’ motivations to donate to a research biobank (motivations for donation to a biobank and reasons for non-participation in biobank research). The third section asked about the type of tissue donated for research purposes. The last section of the questionnaire included questions concerning patients’ demographic characteristics and information relating to their illness.

Previous studies have revealed scant biobank awareness among the Polish population [49–54] so the questionnaire began with the short definition of research biobanks and all the questions used simple descriptive language without technical terminology. All questions were designed as close-ended items offering respondents a limited set of pre-defined and simple answers to choose from. The survey questions were designed to elicit responses on a scale ranging from “Definitely not” to “Definitely yes”, allowing participants to express the strength of their opinions.

### Data collection

The questionnaire was collected from oncology patients of both sexes on five clinical wards at the Institute of Oncology of the University Clinical Hospital in Poznan (the Oncology Ward, the Gynaecological Oncology Ward, the Surgical Oncology Ward, the Clinical and Experimental Oncology Ward and the Chemotherapy Ward) and the Outpatient Clinic and the Division of Gynecological Oncology at the Gynecological and Obstetrics Clinical Hospital of the Poznan University of Medical Sciences.

While a convenience sampling was used in this study, the questionnaires were distributed in-person by two members of the research team (JD and JC) and completed with pen and paper. There was neither monetary nor non-monetary compensation offered to eligible participants to complete the survey. The survey took between about 15 and 20 min to complete.

### Ethical issues

The study followed the ethical standards as laid down in the 1964 Declaration of Helsinki (revised in 2000) [56] and was granted approval by the Poznan University of Medical Sciences Bioethics Committee (KB– 1035/22, granted on 14th December 2022). All patients who volunteered to take part in the survey signed the informed consent form to participate prior completing the survey. The consent form contained information on the aim of the study, research too and the anonymous, voluntary and confidential character of the study. In order to ensure that the participants of the study felt psychologically safe and anonymous the survey was conducted in private and semi-private rooms for patients. Due to the sensitive nature of some questions relating to patients’ illnesses that might cause psychological strain in patients, they were also informed about their right to withdraw voluntarily from the study at any time and for any reason with no consequences. All participants included in the study provided their informed consent to participate.

### Data analysis

Descriptive statistics were employed to summarise participant responses. Counts and percentages were used to present the distribution of responses in Tables 1, 2, 3, 4 and 5. Statistical analysis was conducted using JASP 0.18.1 and significance levels were set at 0.05. No imputation was performed for missing data. For Table 6, stepwise logistic regression analysis was performed in order to identify factors associated with cancer patients’ reasons for participation or non-participation in biobank research. The dependent variable was the likelihood of non-participation, and independent variables included demographic factors, attitudes toward donation and other relevant variables from Table 1. The stepwise selection method was utilised iteratively to include variables that significantly contributed to the model.

In instances of fragmented answers or small respondent groups we consolidated responses to achieve statistical significance. Recognising the significance of religion, respondents were categorised into two groups: those for whom religion held importance and those for whom it was unimportant. Concerning education, participants were similarly divided into two groups based on whether they had higher education. Respondents’ places of residence were categorised into those living in areas with more than 100,000 inhabitants and those with fewer than 100,000 inhabitants. Age groups were established by using the median, creating two categories for those above and below the median.

## Results

Of the 621 cancer patients approached during the five month period 595 completed the survey (response rate 95.8%). However, due to invalid or incorrect answers 55 questionnaires (8.8%) had to be rejected. Thus, in total 548 questionnaires were analysed (92.2% of completed questionnaires). The majority of sample comprised of women (85.2%) (Table 1). Patients’ ages ranged from 19 to 84 with a median age of 52. Most respondents had a university education (52.2%), and lived in areas with populations above 500,000 inhabitants (26.6%). 53% declared as religious.

While respondents represented all stages of cancer, those with intermediate (26.4%), early stage (26.3%) or advanced cancer (24.5%) predominated. 65.5% reported a family history of cancer, and 19.5% genetically determined disease. 20.6% of patients were blood donors and 19.3% were declared bone marrow donors. 83.9% declared being vaccinated against COVID-19.

**Table 1:** Cancer patients’ socio-demographic characteristics

Characteristics	N (%)
*Sex*	
woman	467 (85.2)
man	81 (14.8)
*Patient’s age*	
range	19–84
median	52
IQR (1–3)	43–63
mean (95%CI)	52.46 (51.34–53.58)
SD (95%CI)	13.37 (12.69-14.00)
A*ge at diagnosis*	
range	16–82
median	50
IQR (1–3)	40–60
mean (95%CI)	50(48.86–51.11)
SD (95%CI)	13.37(12.71–14.01)
*Education*	
primary school	7 (1.3)
vocational school	74 (13.6)
high school	170 (33)
university	286 (52.2)
medical university	9 (1.6)
missing	2 (0.3)
*Domicile*	
up to 10,000 inhabitants	149 (27.2)
10–50,000 inhabitants	108 (19.7)
51–100,000 inhabitants	60 (11)
101–500,000 inhabitants	85 (15.5)
above 500,000 inhabitants	146 (26.6)
*What role does religion play in your life?*	
significant	123 (22.5)
rather significant	167 (30.5)
little	146 (26.6)
none	112 (20.4)
*Stage of cancer*	
stage I: non-invasive cancer	59 (10.8)
stage II: early stage	144 (26.3)
stage III: intermediate cancer	145 (26.4)
stage IV: advanced cancer	134 (24.5)
stage V: very advanced cancer	66 (12)
*Is there a history of cancer in your family?*	
yes	359 (65.5)
no	153 (27.9)
I do not know	35 (6.4)
missing	1 (0.2)
*Were there any genetically determined diseases in your family?*	
yes	107 (19.5)
no	220 (40.2)
I do not know	221 (40.3)
*Have you ever donated blood?*	
yes	113 (20.6)
no	434 (79.2)
missing	1 (0.2)
*Are you a declared bone-marrow donor?*	
yes	106 (19.3)
no	437 (79.8)
missing	5 (0.9)
*Are you vaccinated against COVID-19?*	
yes	460 (83.9)
no/prefer not to say	88 (16.1)

Of all patients 43.4% had heard of biobanks, while 56.6% had not (Table 2). Regardless of their biobank (un)awareness 93.1% of respondents declared themselves willing to donate for research purposes. While most patients believed that it was their oncologist (46.3%), followed by other doctors (10.8%) or a surgeon (14%) who should ask patients to share their cancer tissues, respondents suggested that it should be done before the medical procedure, either at the moment of diagnosis (11.1%), pre-operative consultation (23.7%) or before the surgery (26.1%). At same time, 67.7% of patients declared that when biobank researchers conducting research on donated tissue detect information about donor’s disease or genetic predispositions, they should inform both a donor and one’s doctor.

**Table 2:** Biobank awareness among Polish cancer patients

	N (%)
*Have your ever heard of biobanks?*	
yes	238 (43.4)
no	310 (56.6)
*Would you donate your cancer tissues left over after a medical procedure to a biobank for research purposes?*	
definitely yes	331 (60.4)
rather yes	179 (32.7)
rather no	16 (2.9)
definitely no	6 (1.1)
I do not know	16 (2.9)
*Who should ask patients to donate their cancer tissues?*	
my oncologist	254 (46.3)
my doctor should mention it first and other doctors may ask about it	59 (10.8)
a nurse	0 (0)
the surgeon who will perform the procedure	77 (14)
someone from hospital management	2 (0.4)
a hospital representative	16 (2.9)
one of the researchers who will conduct a study using the samples	24 (4.4)
a biobank representative	15 (2.7)
several persons should ask at different stages of cancer treatment and therapy	64 (11.7)
I do not know	35 (6.4)
missing	2 (0.4)
*When is the best moment to ask patients for donation of one’s cancer tissues for research purposes?*	
at the moment of diagnosis	61 (11.1)
during one of the pre-operative consultations	130 (23.7)
before the medical procedure/surgery	143 (26.1)
after the medical procedure/surgery	87 (15.9)
during one of the post-operative consultations	37 (6.8)
when the treatment is over	28 (5.1)
I do not know	62 (11.3)
*What should researchers conducting research on patients’ cancer tissues donated to a biobank do when they discover important health information, i.e. on detection of disease or genetic predispositions*	
nothing	8 (1.5)
inform the donor	104 (19)
inform the donor’s doctor	17 (3.1)
inform both the donor and the donor’s doctor	371 (67.7)
I do not know	47 (8.5)
missing	1 (0.2)

While most cancer patients declared the will to donate blood (80.3%), saliva (66.8%) and hair (65%), almost two-thirds of participants were willing to donate any type of tissue left after a medical procedure (65%), and very few would not donate any type of tissue (4.2%) (Table 3). Additionally, 40.5% of were willing to donate their entire body after their death, while 21% would refuse. Regarding deceased organ donation, patients most frequently mentioned the heart (37.8%), the kidneys (36.5%), the pancreas (30.8%) and the lungs (29.2%).

**Table 3:** The type of tissue donated for research purposes

	N (%)
*Apart from cancer tissue, which of the following tissue would you donate for research purposes?*	
blood	440 (80.3)
nails	263 (48)
skin	198 (36.1)
bone marrow	168 (30.7)
salvia	366 (66.8)
hair	356 (65)
reproductive tissues (sperm, eggs)	108 (19.7)
embryonic cells left after IVF procedure (only women)	65 (13.9)
any type of tissue that is left after the medical procedure	356 (65)
none of the above	23 (4.2)
*Which of the following tissue/organs would you donate for research purposes after death?*	
heart	207 (37.8)
ovaries (only women)	119 (25.5)
intestines	126 (23)
bone	104 (19)
muscle	100 (18.2)
uterus (only women)	113 (24.2)
brain	117 (21.4)
kidney	200 (36.5)
eyes	98 (17.9)
bladder	119 (21.7)
lungs	160 (29.2)
cornea	94 (17.2)
tendon	98 (17.9)
spleen	124 (22.6)
musculoskeletal tissue	95 (17.3)
pancreas	169 (30.8)
liver	192 (35)
ligament	96 (17.5)
heart valve	105 (19.2)
teeth	93 (17)
stomach	150 (27.4)
veins / arteries	97 (17.7)
whole body	222 (40.5)
none of the above	115 (21)

While the vast majority of patients believed that since their cancer tissue had already been taken and may be useful (94.7%) they were primarily driven by altruistic motives (Table 4). These motivations included the desire to help to find a cure for cancer (96.9%), to help others, especially other cancer patients (96.7%) and to help advance research and generate new knowledge on cancer (96.5%). On the other hand, while they stressed that it was important to help others (95.1%), many patients believed that donation might benefit themselves (93.6%) and expected either information about their health status (92.2%) or medical treatment/services (68.4%).

**Table 4:** Cancer patients’ motivations for donation for research purposes

	Definitely not n (%)	Rather not n (%)	I do not know n (%)	Rather yes n (%)	Definitely yes n (%)	Missing
*What would be your primary motivation for donating your cancer tissues for research purposes?*						
to help other people, especially cancer patients	7 (1.3)	0 (0)	11 (2)	45 (8.2)	485 (88.5)	0 (0)
to help myself	9 (1.6)	8 (1.5)	18 (3.3)	48 (8.8)	465 (84.8)	0 (0)
to discover my health status	10 (1.8)	6 (1.1)	27 (4.9)	74 (13.5)	431 (78.7)	0 (0)
to receive medical treatment/services	65 (11.9)	43 (7.8)	65 (11.9)	89 (16.2)	286 (52.2)	0 (0)
to help advance research that might generate new knowledge on cancer	4 (0.7)	2 (0.4)	13 (2.4)	40 (7.3)	489 (89.2)	0 (0)
to help to find a cure for cancer	3 (0.5)	1 (0.2)	13 (2.4)	36 (6.6)	495 (90.3)	0 (0)
to benefit society and future generations	6 (1.1)	4 (0.7)	20 (3.7)	74 (13.5)	443 (80.8)	1 (0.2)
to benefit my family, relatives	3 (0.5)	3 (0.5)	19 (3.5)	37 (6.8)	484 (88.3)	2 (0.4)
I believe it is important to help others	5 (0.9)	3 (0.5)	18 (3.3)	71 (13)	450 (82.1)	1 (0.2)
since my cancer tissue has already been taken, I would like it to be useful	6 (1.1)	6 (1.1)	17 (3.1)	65 (11.9)	454 (82.8)	0 (0)
to maintain good relations with medical personnel	180 (32.8)	51 (9.3)	89 (16.2)	86 (15.7)	142 (25.9)	0 (0)
to receive financial remuneration	371 (67.7)	40 (7.3)	49 (9)	26 (4.7)	61 (11.1)	1 (0.2)

The most common reasons for non-participation in biobank research was related to geographical distance (68.3%) and the need for repeated examinations (63%) (Table 5). Many patients, however, feared that third parties, including insurance companies (50.9%), the government (49.1%) and employers (46.4%) might have access to their samples, or were concerned over the commercial or unethical use of samples (51.1% and 47.1%, respectively).

**Table 5:** Cancer patients’ reasons for non-participation in biobank research

	Definitely not n (%)	Rather not n (%)	I do not know n (%)	Rather yes n (%)	Definitely yes n (%)	Missing
*What are the possible reasons for your refusal to donate cancer tissue to a biobank?*						
geographical distance and the necessity to travel	32 (5.8)	105 (19.2)	37 (6.7)	202 (36.9)	172 (31.4)	0 (0)
the necessity of repeat examinations	30 (5.5)	137 (25)	36 (6.5)	219 (40)	126 (23)	0 (0)
lack of personal benefits from the donation	255 (46.5)	209 (38.1)	48 (8.8)	23 (4.2)	13 (2.4)	0 (0)
lack of financial remuneration	327 (59.7)	156 (28.5)	44 (8)	8 (1.4)	12 (2.2)	1 (0.2)
I think it is a waste of time	347 (63.3)	138 (25.2)	40 (7.3)	13 (2.4)	10 (1.8)	0 (0)
fear over the safety of the data	99 (18.1)	174 (31.7)	45 (8.2)	136 (24.8)	93 (17)	1 (0.2)
it would be against my religious beliefs	317 (57.8)	154 (28.1)	43 (7.9)	15 (2.7)	19 (3.5)	0 (0)
fear over unethical use of the sample	81 (14.8)	174 (31.7)	35 (6.4)	128 (23.4)	130 (23.7)	0 (0)
fear that they will take more samples than necessary	119 (21.7)	239 (43.6)	39 (7.1)	88 (16.1)	63 (11.5)	0 (0)
fear over the invasive nature of the sampling procedure (pain, sight of blood, needles or injections)	89 (16.2)	173 (31.6)	26 (4.7)	125 (22.8)	135 (24.6)	0 (0)
fear of being infected with infectious disease (HIV, sepsis, jaundice)	105 (19.2)	178 (32.5)	31 (5.6)	101 (18.4)	133 (24.3)	0 (0)
fear of detection of disease or genetic predispositions	215 (39.2)	187 (34.1)	51 (9.3)	55 (10)	40 (7.3)	0 (0)
fear that the data generated from the research might result in stigmatisation and discrimination	170 (31)	203 (37)	64 (11.7)	51 (9.3)	60 (11)	0 (0)
fear that my family might know about my health status	307 (56)	171 (31.2)	33 (6)	15 (2.8)	22 (4)	0 (0)
fear over the commercial use of the samples	69 (12.6)	159 (29)	39 (7.1)	106 (19.3)	174 (31.8)	1 (0.2)
fear that the government might have access to the samples	74 (13.5)	153 (27.9)	52 (9.5)	96 (17.5)	173 (31.6)	0 (0)
fear that insurance companies might have the access to the samples	71 (13)	147 (26.8)	51 (9.3)	107 (19.5)	172 (31.4)	0 (0)
fear that employers might have the access to the samples	87 (15.9)	162 (29.5)	45 (8.2)	87 (15.9)	167 (30.5)	0 (0)
I do not trust scientists and such institutions	232 (42.3)	208 (38)	68 (12.4)	11 (2)	29 (5.3)	0 (0)

Biobank awareness did not correlate with cancer patients’ willingness to donate, but factors influencing their attitudes were identified through logistic regression (Table 6). Higher education was associated with greater altruistic willingness (OR = 3.225, *p* = 0.028) and reduced motivation for health status knowledge (OR = 0.491, *p* = 0.039), good relations with medical staff (OR = 0.368, *p* < 0.001), or financial remuneration (OR = 0.344, *p* < 0.001). Younger and religious patients were less interested in exchanging donation for medical services (OR = 1.827, *p* = 0.002; OR = 0.629, *p* = 0.002) and were discouraged by the need for repeat examinations (OR = 1.456, *p* = 0.035). Older patients expressed concerns about insurance companies (OR = 2.012, *p* < 0.001) and employers (OR = 1.755, *p* < 0.001) accessing their samples. Non-religious individuals reported reduced fears about data safety (OR = 0.673, *p* = 0.024), sample misuse against religious beliefs (OR = 0.230, *p* = 0.001), commercial purposes (OR = 0.638, *p* = 0.011), and infection risk (OR = 0.633, *p* = 0.009). Blood donors and older patients had higher concerns about unethical sample use (OR = 1.796, *p* < 0.007; OR = 1.414, *p* = 0.007), and those with a family history of genetic illnesses were more anxious about unnecessary sample collection (OR = 1.755, *p* = 0.011; OR = 1.880, *p* = 0.017).

**Table 6:** Stepwise logistic regression analysis results for factors associated with cancer patients’ attitudes toward biosample donation

Parameter	Estimate	Standard Error	Odds Ratio	Wald Test		
				Wald Statistic	df	*p*
*to help other people, especially cancer patients*						
intercept	2.886	0.285	17.923	102.561	1	< 0.001
higher education	1.171	0.533	3.225	4.817	1	0.028
*to know my health status*						
intercept	2.886	0.285	17.923	102.561	1	< 0.001
patients with higher education	-0.711	0.344	0.491	4.276	1	0.039
*to receive medical treatment/services*						
intercept	0.723	0.166	2.060	18.878	1	< 0.001
younger patients	0.603	0.192	1.827	9.888	1	0.002
religious patients	-0.464	0.190	0.629	5.969	1	0.015
*to maintain good relations with medical personnel*						
intercept	0.046	0.135	1.047	0.115	1	0.734
patients with higher education	-1.000	0.182	0.368	30.320	1	< 0.001
blood donors	0.519	0.221	1.680	5.539	1	0.019
*to receive financial gratification*						
intercept	-1.222	0.152	0.295	64.553	1	< 0.001
patients with higher education	-1.066	0.253	0.344	17.778	1	< 0.001
*the necessity to repeat examination*						
intercept	0.345	0.122	1.412	7.953	1	0.005
younger patients	0.376	0.179	1.456	4.435	1	0.035
*fear over the safety of the data*						
intercept	-0.154	0.119	0.857	1.689	1	0.194
non-religious patients	-0.396	0.176	0.673	5.092	1	0.024
*it would be against my religious beliefs*						
intercept	-2.265	0.202	0.104	125.468	1	< 0.001
non-religious patients	-1.469	0.460	0.230	10.200	1	0.001
*fear over unethical use of the sample*						
intercept	-0.595	0.170	0.552	12.316	1	< 0.001
blood donors	0.586	0.216	1.796	7.374	1	0.007
older patients	0.346	0.176	1.414	3.878	1	0.049
*fear that they will take more samples than needed*						
intercept	-1.325	0.166	0.266	63.762	1	< 0.001
patients unaware of family history of genetically determined disease	0.563	0.220	1.755	6.528	1	0.011
family history of genetically determined disease	0.632	0.265	1.880	5.667	1	0.017
*fear of being infected with infectious disease*						
intercept	-0.077	0.118	0.926	0.421	1	0.516
non-religious patients	-0.457	0.175	0.633	6.813	1	0.009
*fear over the commercial use of the samples*						
intercept	0.044	0.143	1.045	0.094	1	0.760
non-religious patients	-0.450	0.177	0.638	6.433	1	0.011
patients with higher education	0.387	0.178	1.473	4.744	1	0.029
*fear that the government might have access to the samples*						
intercept	-0.407	0.131	0.666	9.677	1	0.002
older patients	0.547	0.174	1.729	9.919	1	0.002
blood donors	0.439	0.215	1.551	4.158	1	0.041
*fear that insurance companies might have the access to the samples*						
intercept	-0.315	0.122	0.730	6.668	1	0.001
older patients	0.699	0.174	2.012	16.114	1	< 0.001
*fear that employers might have the access to the samples*						
intercept	-0.436	0.123	0.647	12.460	1	< 0.001
older patients	0.562	0.174	1.755	10.482	1	0.001

## Discussion

The overall results of this study showed that, although fewer than half of oncology patients had heard of biobanks (43.4%), lack of biobank awareness had no effect on respondents’ affirmative attitudes towards donation for research purposes, as 93.1% of patients declared a willingness to share their HBM with a biobank. These findings corroborate previous studies that showed a broad support for biobank participation and donation among cancer patient, whose willingness to share their biospecimens is much higher than in the general population ranging from 80 to 100% [34–39, 40, 41]. For example, while 84% of cancer outpatients in Australia were willing to share their tissue, and 96% were keen to have their biosamples stored for future research [41], among Eastern Morocco patients it was 80.7% [30] and among British patients 88% [34]. This proportion was even higher among American prostate cancer patients, ranging from 94 to 99% [35, 38].

This study also shows that cancer patients mainly opted for sharing their blood, saliva and hair, but not nails, skin, bone marrow or reproductive tissue. Additionally, although more than 40% of respondents supported post-mortem body donation, they favoured sharing heart, kidneys, pancreas, lungs or stomach. Similar results were found among Chinese patients who declared themselves willing to donate left-over tissue (87.1%) and surplus blood (83.3%) [43]. Similarly, Gao et al. showed that both guardians of children with cancer and adult cancer patients were willing to donate residual blood and marrow (86.5%), urine (85.7%), saliva and faeces (87.5%), extra blood (68.8%) [39]. This, confirms previous research showing that the peoples’ willingness to donate for biobank research is influenced, among other factors, by cultural beliefs about the body and its particular organs. For example, while heart is often perceived as the organic motor of the body or the center of the soul; brain is defined by many as a the essence of humanity and the source of intelligence. Other parts of the body are perceived either as functional (breasts, hands, legs) or aesthetic (face, eyes, breasts, skin, hands) [33, 57–61]. On the other hand, in Polish culture many people believe that human body is an integrated whole than should never be disintegrated or cremated.

Most importantly, this research confirms that cancer patients’ willingness to participate in biobank research is mainly driven by altruism, i.e. the desire to advance science and to help others, especially cancer patients [28, 34, 38, 39, 41, 45, 46, 48]. However, patients in other countries also perceived donation as a moral obligation [46] and stressed that, since their cancer tissues had already been taken, by sharing them they could help advance science and medical progress [34, 39, 40, 43], advance cancer research [28, 38, 42, 44, 46], benefit society and help other people, including future cancer patients [36, 39–41, 45, 46]. This, however, should come as no surprise, since, as cancer patients frequently visit hospitals and depend on the healthcare system and scientific research, they display a high level of trust towards science and doctors. Moreover, during their diagnostic and therapeutic journey they expect close monitoring and high quality treatment, and hope for a cure for their disease. They may therefore perceive scientific research, including that run by biobanks, as more important than does the general public, and may feel obliged to contribute to the development of new diagnostic and therapeutic treatment. They may also perceive donation as an act of gratitude, giving something back, since they had benefited from it themselves during their cancer treatment. Additionally patients’ experience of serious illness may have influenced their desire to be helpful and do something beneficial to society. At the same time, as has been shown by previous studies [34, 39, 48], many cancer patients enrolled in this study perceived donation as an act of reciprocity and expected personal benefits, either in the form of increased knowledge about their health or by receiving medical treatment.

Equally importantly, in accordance with other studies, we found that patients’ declarations regarding participation in biobank research were not unconditional, since donation for biobank research raised many important concerns. While our respondents were mainly concerned with privacy and the safety of their data, unethical use of biospecimens and invasive nature of the sampling procedure, similar risks were also emphasised in previous research where patients voiced doubts regarding privacy and the confidentiality of data, scientific abuse and the possibility of using one’s samples for controversial research such as cloning, or were discouraged by the lack of personal benefit and distrust in biomedical research [28, 32–39]. Many African and White American cancer patients were concerned about the possibility of revealing genetic information about their ethnic or racial group (81%), that their health information might be accessible to their family (88%), employer (78%), healthcare workers uninvolved in the patient’s care (73%) or insurer (63%); and more than half were worried about the possibility of taking too much tissue (67%) [28]. In another study cancer patients raised legal and moral arguments related to immortalisation, commercialisation, scientific abuse for “controversial research” and unconsented use of samples [37, 38]. This research therefore confirms that where patients’ wishes and preferences in terms of consent may be at odds with the needs of science, legal regulations regarding obtaining, using and sharing of HBM and annotated data for research purposes, as well as the issues of consent and ownership of biospecimens are required. Especially, that even though the General Data Protection Regulation have been implemented in Poland by the Personal Data Protection Act of May 2018 which obliges all institutions to protect all personal data and the PBN have created guidelines for data protection [22, 24, 25], still there are no legal regulations regarding biomedical research and biobanking in Poland [14].

Lastly, this research confirms that there are some socio-demographic variables, including age, education level, declared religiousness, being a blood donors and family history of genetically determined disease, which may influence patients’ willingness to share their biospecimens for research purposes [39, 43]. Lee et al., however, also found that older women with a college education, a previous breast biopsy, a family history of breast cancer or a co-morbidity were more likely to donate [45]. In another study by Drake et al. showed that race, a family history of prostate cancer, stage of cancer and grade of cancer significantly linked to patients’ willingness to consent to future use of samples and with their protected health information [35].

### Limitations

This study has a number of limitations that should be acknowledged. Firstly, since cancer patients from only two hospitals in only one city in Poland took part in the study, these results may not express the opinions of patients form other regions and we may be unable to extrapolate our findings to the entire population of cancer patients in Poland. Secondly, the results present responses from only those cancer patients who agreed to complete the survey, so it may not represent the views of those patients who declined to participate. Thirdly, there was implicit gender bias, as female patients outnumbered male patients considerably. However, it must be acknowledged that while the response rate was high (95.8%) the majority of patients admitted to both hospitals during five month of data collection were females. Thus, the unequal proportion of male respondents enrolled in this study results from the structure of patients rather than their unresponsiveness. On the other hand, the gap identified may be due to the fact that even though men get sick more often than women, on average they avoid preventive tests to diagnose cancer, doctors and therapy more often [61–63]. Finally, since many respondents were not biobank unaware, it is possible that they have never considered topics discussed in the survey before and there was a risk of misunderstanding some themes. Additionally, since this study is based on the quantitative method only, to better understand patients’ perspective on such issues as preferred type of consent, autonomy, data protection and confidentiality, control of information and sharing of biosamples, commercialization and profit sharing, which may affect patients’ trust towards biomedical research and willingness to donate to a biobank, further in-depth studies using a qualitative approach are required. At the same time, since to the best of our knowledge, this is one of the first studies on the attitudes of Polish oncology patients towards donation for biobank research, it sheds new light on the topic and may stimulate further research, which may go on to help biobanks in planning and organizing efficient recruitment campaigns among cancer patients.

## Conclusions

This research shows that Polish cancer patients expressed encouraging attitudes towards donating their biospecimens for the purpose of biobank research, and their motivation was driven largely by altruism and their desire to help advance science. It revealed at the same time that there are possible barriers for refusing consent, including geographical distance, fear over privacy and the confidentiality of the data, ethical concerns related to biomedical research, fear over the invasive nature of the sampling procedure and of being infected with infectious disease. Simultaneously, many patients perceived donation in terms of reciprocity and expected compensation for donation, either in the form or personal health information or medical services. They also expressed some preferences on the consent process. To further increase patients’ support for biobanking the following guidelines should be implemented [64–67]:

1. Public awareness campaigns about tissue and organ donation for research purposes should be organised.
2. Posters and information leaflets about biomedical research, biobanks and the role of patients should be prominently placed in various healthcare facilities, so that patients might see them.
3. Healthcare facilities should establish tissue and organ donor co-ordinators trained to identify patients’ who may consent and share their biospecimens. As well as knowledge on the physical and psychological requirements for tissue and organ donation, their training should include the ethical and legal framework for donation, (non-verbal) communication and active listening skills, and knowledge on the role of religious and cultural belief systems on tissue and organ donation.
4. An integrated service platform should be established in order to facilitate better communication between healthy donors, cancer patients and their families, and research institutions.
5. When asking patients to share their biospecimens healthcare professionals should communicate in a patient-centred, supportive, reflective and responsive manner. They should discuss the benefits and risks of the research, address patients’ ethical and moral concerns related to donation and biobanking, and offer resources to help manage these concerns.
6. Since most patients are driven by altruistic motivations and expect no financial remuneration, when asking them to participate, healthcare professionals should explain that donation for biomedical research is a part of something meaningful and significant.
7. Most importantly, although recently the PBN have created new quality standards for Polish biobanks, still there is a urgent need for the development of a legal framework that will regulate the requirements regarding the organization, management and financing of biobanks in the country, the process of obtaining, using and sharing of HBM (both normal and pathologically altered) and clinical data for scientific purposes, and the issue of ownership of biospecimens.

## Data Availability

The datasets generated during and analysed during the current study are available from the corresponding author on reasonable request.

## Footnotes

1 Although some authors suggest that since a person who participates in biobank research does not donate the biological sample but shares it, and is always the owner of one’s biological material and can withdraw consent at any given time, the word “sharing” is more appropriate than “donation”, since both official documents published by the European Union, ISBER or BBMRI and the most literature on biobanks, use the word “donation” we also use it through this paper. Similarly, even though it is sometimes argued that since in the context of biobanking there is not strict donation to other person and for that reason biobank donors should be more appropriately termed “source persons”, following the literature which uses more common term “donor” or “participant” to refer to a person providing one’s biosamples or medical information, to biobanks, through our paper we will also use this term. We are grateful to the anonymous reviewer for bringing our attention to this.

## Abbreviations

BBMRI-ERIC: European Biobanking and BioMolecular resources Research Infrastructure-European Research Infrastructure Consortium
HBM: human biological material
ISBER: International Society for Biological and Environmental Repositories
PBN: the Polish Biobanking Network
PUMS: Poznan University of Medical Sciences
